# A Novel Deep Learning Approach for the Automatic Diagnosis of Acute Appendicitis

**DOI:** 10.3390/jcm13164949

**Published:** 2024-08-22

**Authors:** Kamil Dogan, Turab Selcuk

**Affiliations:** 1 Radiology Department, Faculty of Medicine, Kahramanmaras Sutcu Imam University, Kahramanmaras 46050, Turkey; 2 Department of Electrical and Electronics Engineering, Kahramanmaras Sutcu Imam University, Kahramanmaras 46050, Turkey; turabselcuk23@gmail.com

**Keywords:** acute appendicitis, computed tomography, artificial intelligence

## Abstract

**Background:** Acute appendicitis (AA) is a major cause of acute abdominal pain requiring surgical intervention. Approximately 20% of AA cases are diagnosed neither early nor accurately, leading to an increased risk of appendiceal perforation and postoperative sequelae. AA can be identified with good accuracy using computed tomography (CT). However, some studies have found that a false-negative AA diagnosis made using CT can cause surgical therapy to be delayed. Deep learning experiments are aimed at minimizing false-negative diagnoses. However, the success rates reported in these studies are far from 100%. In addition, the methods used to divide patients into groups do not adequately reflect situations in which accurate radiological diagnosis is difficult. Therefore, in this study, we propose a novel deep-learning approach for the automatic diagnosis of AA using CT based on establishing a new strategy for classification according to the difficulties encountered in radiological diagnosis. **Methods:** A total of 266 patients with a pathological diagnosis of AA who underwent appendectomy were divided into two groups based on CT images and radiology reports. A deep learning analysis was performed on the CT images and clinical and laboratory parameters that contributed to the diagnosis of both the patient and age- and sex-adjusted control groups. **Results:** The deep learning diagnosis success rate was 96% for the group with advanced radiological findings and 83.3% for the group with radiologically suspicious findings that could be considered normal. **Conclusions:** Using deep learning, successful results can be achieved in cases in which the appendix diameter has not increased significantly and there is no significant edema effect.

## 1. Introduction

Acute appendicitis (AA) is a major cause of acute abdominal pain requiring surgical intervention and is prevalent among individuals aged 20–30 years [1]. In emergency departments with high patient congestion, the aim is to swiftly and effectively diagnose or rule out AA. Approximately 20% of AA cases are diagnosed neither early nor accurately, leading to an increased risk of appendiceal perforation and postoperative complications [2]. Appendectomy is the definitive treatment for AA. Morbidity is 100 times higher in cases of appendiceal perforation than in cases of simple AA, with corresponding mortality rates of 10% vs. 0.1%. Therefore, early diagnosis and surgical intervention are crucial [3].

Anamnesis and physical examination are the most important steps in the diagnosis of AA [4]. In 1986, Alfredo Alvarado developed a 10-point scoring system called MANTRELS, which is based on clinical and laboratory findings, to diagnose AA in symptomatic patients. The scoring system recommended that patients with ≥7 points should undergo surgery, whereas those with <7 points should be followed. However, the Alvarado scoring system results in increased complications, mortality, and morbidity in older patients with AA owing to inaccurate and delayed diagnosis [5].

In most patients, diagnosis can be made through clinical observations and laboratory tests. Despite the high rate of negative appendectomy diagnoses in procedures based on medical history, physical examination, and test results, delays in atypical cases can lead to major consequences such as abscess, perforation, peritonitis, and plastron in 4–15% of all cases. Therefore, more efficient techniques are required for early and accurate diagnoses [6].

The most preferred imaging tool for diagnosing AA is ultrasonography (US), although magnetic resonance imaging (MRI) is considered equally essential [7]. The diagnosis of AA using computed tomography (CT) is fairly accurate, with a rate ranging from 93% to 98%. Furthermore, some studies have found that enhanced-contrast CT has greater sensitivity than non-contrast CT in diagnosing AA, supporting the use of contrast material. CT findings relevant to AA include an enlarged appendix (>6 mm in diameter), enhanced contrast material on the appendiceal wall, indistinguishable wall borders, and the presence of appendicoliths. Peri-appendiceal findings may involve thickening of the cecal wall (>3 mm), increased density due to fatty tissue inflammation, and the presence of inflammatory collections. These findings can be categorized into two groups depending on symptom presentation. In 42% of patients who are asymptomatic, the appendiceal diameter exceeds 6 mm, which hinders its use in diagnosing AA. In addition, a comparable increase in the appendiceal diameter is observed in cases of ovarian cyst rupture and inflammatory bowel illness. The high sensitivity and specificity of CT make it crucial for therapeutic planning. CT has been found to positively impact diagnosis and/or treatment for 45.6% of patients [8], decreasing the rate of unnecessary appendectomies, particularly in cases of uncertain diagnosis. The negative appendectomy rate is 16–24% without CT but only 5% with CT [6]. Coursey et al. found that CT decreases the rate of unnecessary appendectomies only in female patients aged <45 years, whereas the rate is comparable between males and older women [9]. The high sensitivity of peri-appendiceal inflammation, which is enhanced by pericecal adipose tissue acting as an intrinsic contrast agent, may contribute to variations in the diagnosis of appendicitis among different sexes and age groups. A review of the effect of obesity on postoperative outcomes in children undergoing appendectomy for AA concluded that this effect is unclear; therefore, it is not possible to draw a confident conclusion with the available data [10]. Furthermore, it reduces treatment expenses by preventing unnecessary surgeries in addition to enabling the distinction between appendiceal abscesses and epiploic appendicitis. An appendiceal abscess is managed with surgery, whereas epiploic appendicitis is treated with medication [11]. Therefore, a more accurate diagnosis of AA is required. The surgeon initiates surgery based on an accurate diagnosis, facilitating patient consent. This assists in identifying the precise location for surgical incision. Precisely managing the affected area in cases of perforated appendicitis is important because it reduces the incidence of perforated appendicitis by preventing unnecessary follow-up appointments and wasted time. This helps in diagnosing cases in which AA is undetectable using US [12] and is also useful for identifying abnormalities in the abdomen [4,13].

Artificial intelligence (AI) has tremendous potential to revolutionize and accelerate the development of diagnostics and treatments in regenerative medicine. From improving drug discovery to optimizing tissue engineering and cellular therapies, AI can provide insights by analyzing large molecular and genomic datasets that would be impossible for humans to understand [14]. On the other hand, there is a need to ensure that learning models are interpretable. Higher model interpretability means that future predictions are easier to understand and explain to end users. Furthermore, interpretable learning models enable healthcare professionals to make reasoned and data-driven decisions to provide personalized decisions that can ultimately lead to higher-quality healthcare. In general, interpretability approaches can be divided into two groups: the first focuses on personalized interpretation (local interpretability), and the second summarizes predictive models at the population level (global interpretability) [15].

There are numerous recent studies demonstrating the significant advancements made in using artificial intelligence (AI) for detecting AA. Deep learning (DL) and image processing techniques have emerged as promising approaches in this field. DL algorithms have demonstrated heightened sensitivity and specificity in diagnosing appendicitis based on ultrasound images compared to expert clinicians. In one study, a machine learning model was used to predict perforated and non-perforated AA. The study utilized data from 1797 patients, achieving an accuracy rate of 88.2% in distinguishing AA from other conditions. Moreover, the accuracy rate was 92% for differentiating between perforated and non-perforated cases. The researchers employed explainable AI methods to highlight important biochemical markers, making the model’s predictions more transparent and understandable [13].

In another study, a novel approach was introduced for differentiating between complicated and uncomplicated cases of AA. The researchers aimed to enhance diagnostic accuracy by integrating DL and radiomics. DL was employed to extract relevant features from medical images, while radiomics provided additional quantitative and qualitative data. The combined model demonstrated promising results in differentiating between the two types of appendicitis, suggesting its potential for improving clinical decision making [16,17]. Studies have been conducted on the diagnosis of AA using CT and deep learning techniques, where the focus was on using images [18] where radiologists had diagnosed AA with confidence. However, in certain images, radiologists could not make a definitive classification as normal or AA. Within this group, some exhibited an intermediate diameter at the point where the normal and pathological groups overlapped, and there were instances when inflammation in the peri-appendiceal fatty tissue was subtle, which hindered identification. This is the group specifically targeted in our study. In addition, studies have indicated the existence of a group in which the appendix is not visible on CT. In our study, we partially characterize this group and offer suggestions.

This study investigates the integration of a hybrid Convolutional Neural Network (CNN) model with ensemble learning techniques to improve the detection of acute appendicitis from CT images. The hybrid CNN models used are DenseNet, VGG16, and MobileNet; these models are combined with ensemble learning classifiers, including SVM, KNN, and Random Forest. The novelty of this work lies in the significant enhancement of detection performance achieved by integrating these models and classifiers with a comprehensive feature vector that includes clinical, radiological, and biochemical features. This approach provides a meaningful improvement over existing methods, demonstrating that advanced techniques can offer higher diagnostic accuracy.

## 2. Materials and Methods

### 2.1. Ethical Approval and Informed Consent

The study was approved by the ethics committee of the local university hospital (decision number: 2020/17) and conducted in accordance with the principles outlined in the Declaration of Helsinki. In this retrospective study, CT images were analyzed on PACS.

### 2.2. Patients

Patients included in this study were those with a preliminary diagnosis of AA who underwent surgery at the Kahramanmaraş Sütçü İmam University School of Medicine and whose diagnosis was pathologically confirmed as AA. Patients who underwent preoperative contrast-enhanced abdominal CT were eligible for inclusion. There were 6 patients who had AA but were not observed by a radiologist, meaning they had negative CT scans. These patients were excluded from the analysis due to insufficient data. In these patients, the appendix was also superimposed with bowel loops. Wall thickness and surrounding edema could not be identified. They were considered a group and suggested for future studies. Despite the varying degrees of superimposition, those with observed wall thickness and surrounding edema were included in Group 1 or Group 2. The control group consisted of an equal number of participants as in the patient group and was standardized based on the patients’ age and sex data. The patients were initially categorized into three groups. However, one group was omitted owing to the insufficient number of patients in this group. Thus, this study involved images from two groups of patients, 266 in total (101 females and 165 males), aged 13–68 years. Similarly, a control group containing 266 individuals (110 males and 156 females) was created. The control group had the same age distribution as the patient group. Presented in Table 1 are the radiological, laboratory, and clinical data of Groups 1 and 2 and the control group.

**Figure 1:**
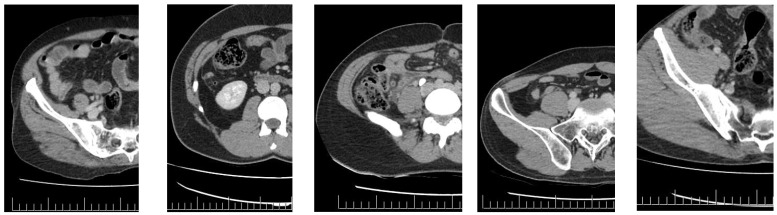
Study criteria for Group 1 (<9 mm diameter and absence of obvious edema).

**Figure 2:**
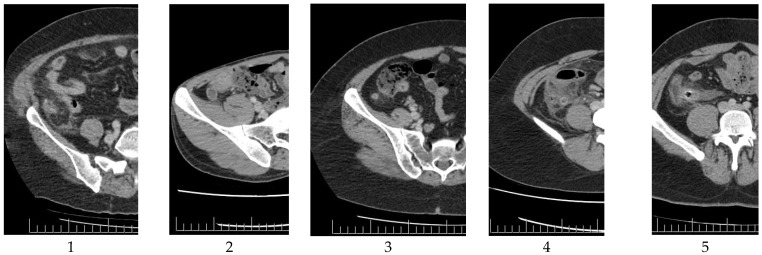
Study criteria for Group 2 (**Left** to **right**: 1. Significant edema with increased diameter; 2. Increased diameter without obvious edema; 3. Significant edema without a significant increase in diameter; 4. Slight increase in diameter with significant edema; and 5. Significant edema, increased diameter, and presence of fecaloid).

Group 1 included 66 patients, whereas Group 2 included 200 patients. The study criteria for Groups 1 and 2 are illustrated in Figure 1 and Figure 2, respectively.

### 2.3. Clinical and Laboratory Parameters

Patients commonly presented with right lower quadrant pain, fever, increased leukocyte counts, and elevated C-reactive protein (CRP) levels.

### 2.4. Significant Parameters in the Control Group

The control group was standardized based on patients’ age and sex. Participants in the control group showed no pathological findings in the right lower quadrant on CT. Furthermore, their CRP levels and leukocyte counts were within normal ranges. For the control group, the anamnesis and physical examination findings were disregarded.

### 2.5. Computer-Aided Deep Learning Method

In this study, a hybrid convolutional neural network (CNN) model was developed using ensemble learning. In the Feature Extraction stage CT images were input into VGG16, MobileNet, and DenseNet models, which were pre-trained on the ImageNet dataset. Each model extracted high-level features from the images. During feature extraction, the 1 × 1000-dimensional features obtained from the fully connected layers of each CNN model were combined, resulting in a deep feature vector (DF) with dimensions of 1 × 3000.

In the Feature selection stage, to reduce the dimensionality of the comprehensive feature vector and improve performance, the Minimum Redundancy Maximum Relevance (MRMR) algorithm was used. MRMR selected the most relevant 1000 features with minimal redundancy, ensuring the model operated more efficiently and effectively. The number of 1000 features was determined manually. 

In the Feature Combination stage, the feature vectors obtained from VGG16, MobileNet, and DenseNet were combined to create a comprehensive feature vector. This combined vector aimed to provide a richer representation by integrating different perspectives offered by each model. Additionally, features obtained from clinical findings, radiological findings, and biochemical analyses were included in the comprehensive feature vector. Features from radiological findings include the presence of fecaloid and edema, labeled as Rf1 and Rf2, respectively. Features from clinical findings indicate the presence or absence of defense and rebound tenderness, labeled as Cf1 and Cf2, respectively. Features from biochemical analyses include C-reactive protein (CRP) and white blood cell count (WBC), labeled as Bf1 and Bf2, respectively. As a result, a feature vector with dimensions of 1 × 1006 was created, consisting of 1000 deep features (DF), 2 clinical features (CF), 2 radiological features (RF), and 2 biochemical features.

In the classification stage, to further enhance the performance of the classification model, ensemble learning was utilized. Ensemble learning has been shown to significantly enhance performance by effectively combining multiple models, a concept well-supported by numerous studies that highlight its widespread application and effectiveness in improving diagnostic accuracy [19]. In this study, an ensemble learning approach was created that includes SVM, KNN, and Random Forest classifiers. In Weight Assignment, the weights were determined based on the classifiers’ individual performance, such as accuracy or other relevant metrics. Classifiers that demonstrated higher performance were assigned greater weights. In the voting process, for each prediction, the output from each classifier was multiplied by its respective weight. These weighted predictions were then summed, and the final classification decision was made based on the weighted average.

**Figure 3:**
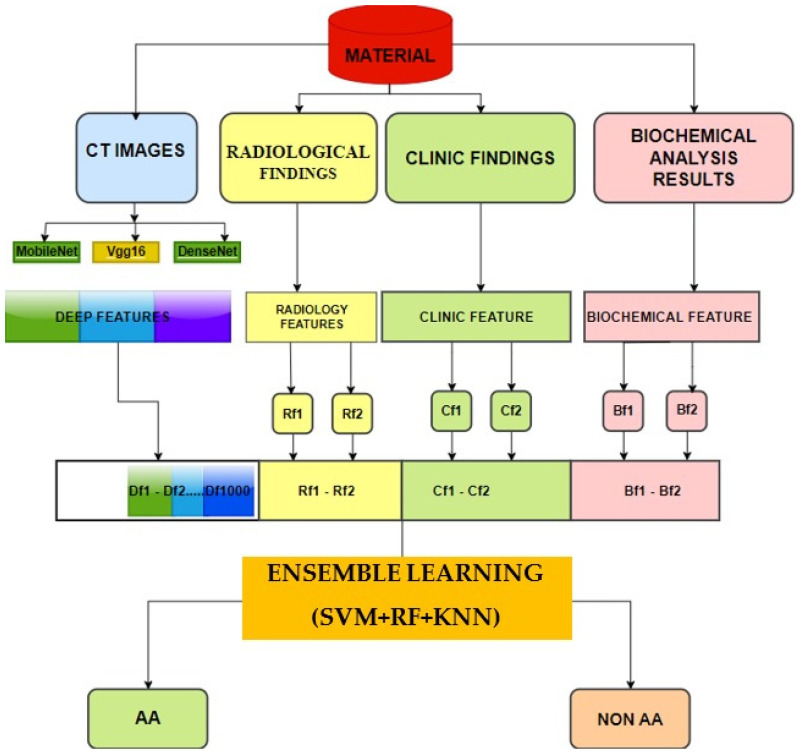
Flowchart of the computer-aided deep learning method. CT—computed tomography; AA—acute appendicitis; SVM—support vector machine; RF—random forest; KNN—k-nearest neighbor.

This ensemble approach aimed to leverage the strengths of each classifier while mitigating their individual weaknesses, leading to a more robust and accurate classification outcome. A flowchart including these techniques is shown in Figure 3.

**Figure 4:**
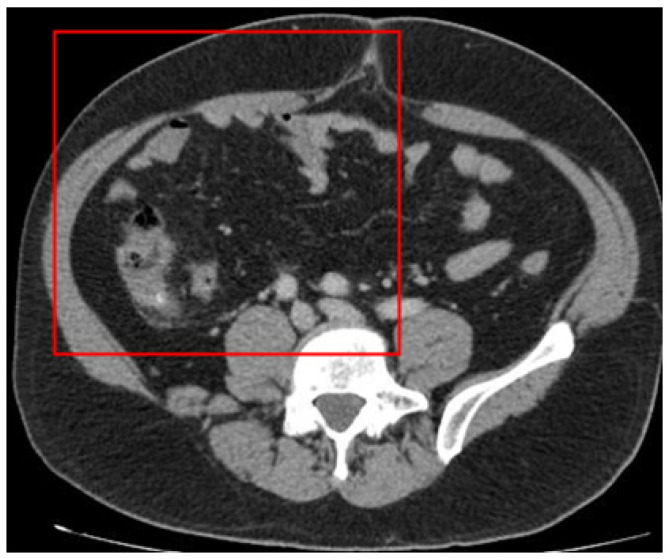
The image section used for deep learning (the red square).

We focused on the region where the appendix is typically situated, which is the right side of the body (left in the figure). Raw images were partitioned into four sections according to the Cartesian plane system. The appendix in the dataset images is situated in the upper-left section and measures 250 × 600 pixels. Appendicitis was excluded from other regions where the appendix could not be located. The image size was therefore reduced from 1255 × 515 pixels to 250 × 600 pixels for analysis (Figure 4).

### 2.6. Model Analysis

We used 70% of the dataset (186 patients and 186 controls) for training and 30% (80 patients and 80 controls) for testing. A confusion matrix was used to examine the model. True positive refers to the number of cases correctly classified as AA, false positive refers to the number of cases that were not AA but were misclassified as AA, true negative (TN) refers to the number of cases correctly classified as not AA, and false negative (FN) refers to the number of cases that were AA but were not classified as AA. Equations (1)–(4) show the formulas for calculating sensitivity, specificity (recall), precision, and the F1 score, respectively.

(1) $$Sensitivity=\frac{TP}{TP+FN}\times 100\%$$

(2) $$Specificity=\frac{TN}{TN+FP}\times 100\%$$

(3) $$Precision=\frac{TP}{TP+FP}\times 100\%$$

(4) $$F1=\frac{2\times TP}{2\times TP+FN+FP}$$

## 3. Results

The performance of the deep learning-based AA diagnosis model was assessed using comparative analysis. Table 2 presents the results of the three CNN models and the hybrid model in which they are integrated. Artificial intelligence proved to be successful in diagnosing AA as demonstrated by the hybrid model, which is based on a combination of the VGG16, DenseNet, and MobileNet models. The sensitivity indicates the model’s ability to detect AA, and the sensitivity values for MobileNet, VGG16, and DenseNet were 91.4%, 92.3%, and 91.7%, respectively. The hybrid model with the SVM setup achieved a sensitivity of 94.1%. In this study, we found that the sensitivity of the ensemble learning categorization based on decisions made by several classifiers was 95.7%.

Specificity is the ability to correctly identify cases that were not AA. The proposed model achieved a specificity of 69.7%. This value is lower than the sensitivity, precision, and F1 score values because of the similarities between specific AA and non-AA images in Group 2. It is expected that additional data from Group 1 and individuals without AA would enhance the specificity.

Table 3 presents the accuracy of appendicitis (AA) diagnosis in Groups 1 and 2. Of the 66 patients in Group 1, 55 were true positives (TP) and 11 were false negatives (FN). In Group 2, out of 200 patients, 192 were true positives (TP) and 8 were false negatives (FN). Specifically, false negatives in the hybrid model were 13 for Group 1 and 9 for Group 2. However, with the implementation of ensemble learning, there was a reduction in false negatives. Additionally, the proportion of patients in Group 2 was higher than in other groups, and radiologists had no difficulty in making diagnoses. In Group 1, the patient proportion was lower, posing challenges for radiologists in diagnosing them, although deep learning methods remained effective.

## 4. Discussion

The unique classification and deep learning system in this study, which utilizes anamnesis, physical examination, laboratory, and imaging data, outperformed existing methods in diagnosing AA. The diverse background, sensitivity, and specificity data crucial for the successful diagnosis of AA are outlined in this study. The high success rate of sensitivity starkly contrasts with the minimal improvement in specificity, which may be attributed to the constraints related to the selection of the control group. Alternatively, this may be attributed to other factors, such as the emphasis on numerous differential diagnostic alternatives in this study. An in-depth examination of the established diagnostic criteria for AA outlined in the literature would clarify the relationship between these criteria and the data from this study.

### 4.1. A Look at the Study According to the Important Points in AA Diagnosis in Clinical Practice

#### 4.1.1. Anamnesis and Physical Examinations

Anamnesis and physical examinations are crucial in diagnosing AA. In this study, anamnesis and physical examination data were collected, including symptoms such as abdominal discomfort, temperature, defense, and rebound in the right lower quadrant. However, these parameters were universal in all patients but not assessed in the control group. Describing criteria, such as stomach discomfort, fever, defense, and rebound, as either present or absent is therefore a limitation that may result in different interpretations.

#### 4.1.2. Laboratory Results

According to the laboratory results, all patients exhibited elevated leukocyte counts and CRP levels, whereas the control group comprised individuals with standard levels. Although this approach resulted in precise conclusions, it also posed constraints. Studies that include control group cases with elevated leukocyte counts and CRP levels may provide valuable information, as both markers increase in many types of inflammation. One limitation of this study is that information about the levels of certain parameters was included, but the relationships between these different levels were not explored.

#### 4.1.3. Patient Age

Studies have discussed how patient age affects the ease or complexity of diagnosis. This study included patients of a wide range of ages, from children to older individuals. Thus, it had the advantage of reporting a high success rate in a patient population representing a wide age range. In future studies, further analysis of the differences among age groups should be conducted for categorizing these groups. At our center, pediatric patients are mostly diagnosed using anamnesis and physical examination. When radiography is necessary, the US is the preferred imaging modality, with CT being performed in a few patients. Thus, the corresponding number of pediatric patients was very low (the number of patients aged <18 was one in Group 1 and five in Group 2).

#### 4.1.4. Patient’s Sex

The diagnostic success of CT is influenced by the patient’s sex [6,9,20]. Although we indicated the patient sex in this study, the diagnostic success was not assessed on the basis of patient sex.

#### 4.1.5. Radiological Imaging

In cases of suspected AA, imaging approaches are more effective for accurate diagnosis than analyzing medical history, physical examinations, laboratory results, or scores [21,22]. Notably, the US is the most preferred imaging technique [23], while MRI is also crucial for diagnosing AA [7]. CT is highly accurate in diagnosing appendicitis, with an accuracy rate of 93–98%; however, it has certain established limitations [4,9,24]. These limitations are contingent on the patient, CT images, or the radiologist’s interpretation. From the radiologist’s perspective, we have achieved significant success using deep learning. From the patient’s perspective, intestinal superposition may contribute to limitations. The number of patients included in this study was limited.

Previous studies have indicated that the use of intravenous contrast material in CT improves the diagnosis of abdominal aortic aneurysms. Contrast-enhanced CT has demonstrated higher sensitivity than non-contrast CT, suggesting the use of contrast material is beneficial. In this study, only contrast-enhanced CT images were assessed. Future research including non-contrast CT is necessary for patients for whom contrast materials cannot be used.

### 4.2. Overview of Study Groups and AA Diagnostic Parameters in These Groups

The study groups were selected to align with the primary goal of addressing the challenges in AA diagnosis during routine radiography. The groups were established according to the three cases of AA diagnosis observed during routine radiological practice: (1) cases that the radiologists deemed normal or could not definitively diagnose despite a preliminary clinical diagnosis; (2) cases of diagnosis confirmed by a radiologist; and (3) cases in which radiologists encountered challenges in interpreting or identifying the appendix because of superimposition.

#### 4.2.1. Group 1

Group 1 included cases with borderline and moderate appendiceal double-wall thickness, ambiguous peri-appendiceal edema indicating inflammation, and no appendicoliths. No radiologist reported a conclusive diagnosis of AA based on the patient’s CT image. Notably, some reports omitted the appendix, some stated that it was normal, and others had ambiguous references, such as possible signs of appendicitis, and advised further examinations. Group 1 included cases in which the radiologists were uncertain about the presence of AA, highlighting the importance of this study. Multiple radiologist remarks, such as a normal appendix and suspicion of AA, were grouped for analysis. Subsequent research should consider analyzing these reports individually.

##### Appendiceal Diameter

The appendiceal diameter was assessed in this study as follows: in the control group, the maximum appendiceal double-wall thickness was 10 mm. The threshold was set to 9 mm because only a few participants in the control group had a diameter of 10 mm. Briefly, the appendiceal diameter did not serve as a distinguishing parameter in this group because the double-wall thickness ranged from 6 mm to 9 mm in both the patient and control groups. Appendiceal diameter is a straightforward sonographic criterion that exhibits good agreement among radiologists in cases of suspected AA [25]. Notably, some studies have found that the appendiceal diameter is not influenced by age and can exceed 6 mm in 42% of asymptomatic patients, hindering its effectiveness in diagnosing AA. Some studies also indicate that 23% of adult men have appendages that are healthy in terms of the cecum and larger than 6 mm in diameter. Therefore, they suggest that when an appendix is found with a diameter between 6 and 9 mm, it should be considered “undetermined” and other findings of AA should be investigated [26]. Conversely, it has been reported that the appendiceal diameter does not play a major role in ruling out complex appendicitis [27]. The results for appendiceal diameter in this study are consistent with those of previous studies. Furthermore, a comparable increase in the appendiceal diameter may be observed in cases of ovarian cyst rupture and inflammatory bowel disease. In this study, we deliberately excluded patients with different primary conditions in the right lower quadrant. We identified patients with AA based on comparison with a healthy control group. Future research on this quadrant should aim at distinguishing between AA and other disorders.

##### Pericecal Fat Tissue

Pericecal fat tissue acts as an inherent contrast agent, making peri-appendiceal inflammation the most notable finding on non-contrast CT, with a sensitivity of 98–100%. This criterion was prioritized in our classification because of this significance. Edema in this region was assessed as a basis for classification into Group 1, and the subjectivity involved with assessing peri-appendiceal inflammation was addressed through the establishment of three quantifiable criteria: (1) having thinner edema compared to the thickness of the single appendiceal wall or the opposing wall; (2) restricted distribution of edema throughout the wall; and (3) edema not entirely encircling the wall. If at least one of these criteria was met, the edema was classified as mild, and the patient was placed in Group 1. These criteria were helpful in providing some level of objectivity; however, they were still based on personal opinions. For example, widespread occurrence is indicated in the second requirement. These criteria could be more explicitly articulated.

The success rate of deep learning for Group 1 diagnosis was 83.3%, which is relatively high. However, this approach did not result in diagnosis for a substantial proportion of cases. Fewer patients were included in this group than in the other groups. Increasing the number of cases and studies using various methodologies could enhance the diagnosis rate.

#### 4.2.2. Group 2

Group 2 consisted of cases that could be identified as AA by any radiologist based on CT. All patients in this group had CT findings that clearly indicated AA. The radiologists did not encounter any diagnostic challenges with this patient cohort.

Within this group, the following CT imaging criteria were established: appendiceal double-wall thickness ≥10 mm, wall thickness ≥6 mm, considerable peripheral inflammation, or the existence of appendicoliths.

##### Appendicoliths

This study focused only on the significance of the presence of appendicoliths and did not consider modifications in the diameter and lumen before and after nor the specific characteristics of the appendicolith, such as size, count, or position within the appendix. For all patients, identification was based on the presence of appendicoliths.

The success rate of AA diagnosis using deep learning was 96%. However, this rate should be increased to 100%. This rate can be enhanced by increasing the number of cases and/or studies conducted using various methodologies.

#### 4.2.3. Group 3

There was also a third group of patients. The inclusion criteria for this group were as follows: (1) the fat tissue around the appendix could not be seen due to bowel superposition (in this case, an edema assessment could not be made). (2) The appendix wall could not be clearly defined due to bowel superposition (in this case, the wall thickness could not be clearly assessed). Moreover, in this group, radiologists could not precisely localize the appendix. Therefore, they could not comment on the appendix. Patients with these characteristics were collected in one group. However, since there were only six patients in this group, they were excluded from the study because it was thought that it would not be sufficient for analysis. However, we wanted to remind the researchers that artificial intelligence studies conducted with these cases, which present radiologists with a high degree of difficulty in making a diagnosis, would perhaps represent the most advanced level in the diagnosis of AA.

### 4.3. Some Other Situations in AA

#### 4.3.1. Uncomplicated Appendicitis

Recent findings have indicated that antibiotic [28] or probiotic [29] treatment may serve as a substitute for surgery in patients with uncomplicated appendicitis. Patients were selected for antibiotic treatment on the basis of having a straightforward finding of AA on CT. A previous study [27] focused on distinguishing between patients with and without complications using deep learning algorithms. However, in this study, this distinction varied. The patients in Group 1 had no complications, whereas those in Group 2 exhibited radiographic signs of appendicitis. However, these patients were not categorized based on the presence or absence of complications. In this study, we aimed to conduct identification in the group that exhibited substandard radiological results. This group may be the most crucial segment, without any difficulties, and expected to mainly benefit from antibiotic treatment. Notably, our groups may also be skewed regarding selection for antibiotic therapy. In addition, interaction between specialties (pediatric surgeons, pediatricians, radiologists, microbiologists, infectious disease specialists, and molecular biologists) should be a prerequisite in the diagnosis, treatment, and management of AA in order to increase success in the fight against bacteria. The era of accepting a single decision-making physician as the authority should be a thing of the past [28].

#### 4.3.2. Perforated Appendicitis

A wider control region is necessary in cases of perforated appendicitis. In such cases, the surgeon’s choice of incision location is crucial [6]. This indicates that basic diagnosis would be inadequate, and perforation data should therefore be included. In this study, perforated cases were not separately categorized but included in the group with a definitive diagnosis of AA (Group 2).

#### 4.3.3. Epiploic Appendicitis, and Mesenteric Lymphadenopathy

Differentiating between AA, epiploic appendicitis, and mesenteric lymphadenopathy is crucial. AA can be surgically addressed, whereas the other conditions can be treated medically. However, this distinction was omitted from this study. We ensured that patients with epiploic appendicitis and mesenteric lymphadenopathy were excluded from the control group. Thus, these differences should be incorporated into future studies.

#### 4.3.4. Location of the Appendix

In this study, the location of the appendix (for example, retrocecal) and the direction in which the findings extended in the abdomen were not specified. These findings may extend in multiple directions, including medial, lateral, inferior, and proximal. Such information may enhance the surgeon’s preoperative preparation [30]. In this study, diagnostic accuracy was deemed to be substantially independent of the direction of extension. The success rate was acceptably high regardless of the direction of the appendage tip. As the direction was not specified, the findings cannot contribute to surgical orientation.

#### 4.3.5. Pathological Findings

The strength of our study is that all trial data were validated based on pathological findings. However, the pathological subgroups were not specified, which is another limitation.

### 4.4. A Look at the Study from the Perspective of Deep Learning Methods

Our proposed method outperformed existing deep learning methods in terms of all parameters, underscoring the significance of method selection.

While applying this approach, the bottom-right quadrant, where the appendix is located, was accurately marked as the primary position of the appendix. Marking the position may limit the opportunity to identify potential atypical locations of appendiceal extension, particularly at the tip. The limited variety and number of our CT images may affect the generalization ability of the model. Data obtained from different centers to increase the data variety may increase the performance of the model. Another limitation is the use of pre-trained models to avoid time-consuming and high computational requirements. However, retraining the model with the existing dataset can significantly improve the performance. Another limitation is that the presence of AA is investigated in a manually selected region from the right quadrant of the CT images. The fact that AAs outside this region cannot be detected can be considered as another deficiency.

Hybrid CNN models consolidate the strengths of different architectures to achieve higher accuracy rates and better generalization capabilities. A hybrid CNN model can achieve higher accuracy in medical image classification compared to individually using the component models [31]. Additionally, hybrid models become more resilient to noise by integrating information from various data sources. This is particularly crucial in reducing the impact of noise and artifacts in medical images. The combination of different CNN layers allows for the extraction of richer and more distinctive features, which is especially useful in detecting complex structures and details. In one study, a hybrid CNN-SVM model was proposed for classifying lung cancer medical images. By combining a convolutional neural network (CNN) for feature extraction and a support vector machine (SVM) for classification, it was observed that the model had improved accuracy in lung cancer diagnosis [32].

A similar phenomenon applies in this study. The proposed hybrid model consists of the combination of three different CNN models. Although each of these models individually achieves high performance, the proposed hybrid model leads to a significant improvement in the detection of AA.

In this study, we focused on the region in which acute appendicitis is commonly observed. We achieved a high success rate in detecting AA within this region. However, our model may fail to detect possible appendicitis cases outside of this region. Therefore, it provides local and limited reliability [33]. We examined interquartile range (IQR) values to observe the effect of outliers in the dataset. IQR is a method used in statistics to observe the impact of outliers or extreme values in a dataset. When the obtained IQR values and standard deviation values were examined together, the wall thickness, WBC, and CRP values showed consistency with each other. The other focus of this study was to extract maximum features from CT images to describe AA. The outcome of this study was significantly influenced by CT data. In Group 1, 20 patients were categorized as normal and 28 patients as suspicious by the radiologist. Therefore, the AA detection performance in Group 1 was lower compared to Group 2. Some patients in Group 1 showed similarities in WBC values to the control group, which could negatively affect the system’s performance. However, due to the structure of CNN models, increasing the amount of data will reduce the impact of this similarity on performance over time.

The performances of CNN models and the accuracy and reliability of these performances are directly associated with the dataset. Two primary approaches were used to determine the accuracy of the model: holdout validation and cross-validation. In holdout validation, the dataset is divided into training and testing sets in a certain proportion, while in cross-validation, the entire dataset undergoes both training and testing by changing the test data each time. Since cross-validation is a more time-consuming process, holdout validation was used in this study. However, for the reliability of the proposed model, it is beneficial to implement both methods [34].

As can be seen in Table 2, the model’s performance metrics, including its sensitivity, specificity, precision, and F1 score, were obtained separately. Sensitivity measures the accuracy of the model’s positive predictions. Specificity indicates the model’s accuracy in identifying negative examples and is usually evaluated in tandem with sensitivity. High specificity means that the model has a low false positive rate and can correctly distinguish negative examples [35]. Precision shows how many of the positive predictions are actually positive. The F1 score represents the balance between precision and sensitivity. To accurately evaluate a model, these four scores need to be examined together. MobileNet exhibits the highest performance in terms of sensitivity (91.4%), but it has lower specificity and accuracy compared to other models. VGG16 provides slightly higher sensitivity (92.3%) and accuracy (88.7%) but lower specificity than MobileNet. DenseNet demonstrates a similar performance to MobileNet and VGG16 in terms of sensitivity and accuracy, but it has lower specificity. Based on a comparison, the hybrid model has the highest sensitivity (94.1%) and accuracy (89.4%). However, its specificity still lags. The Hybrid+ EL (proposed) model demonstrates the highest performance across all metrics, achieving the highest sensitivity (95.7%), specificity (69.7%), accuracy (92.8%), and F1 score (94.2%). The Hybrid+ EL model provides a balanced performance, yielding generally better results compared to other models. In conclusion, this study demonstrates that hybrid models can provide more successful results compared to singular models. In this regard, it serves as a basis for future research. However, to generalize the accuracy of the model further, data from different centers are required. Additionally, employing cross-validation can enhance the model’s performance and accuracy. In an upcoming study, data will be collected from Group 3, which consists of cases in which it is nearly impossible for a radiologist to distinguish AA using CT scans. A new detection system will be developed based on these data.

## 5. Conclusions

The accurate diagnosis of AA is important for early surgical intervention and to avoid complications. For this purpose, the possibility of using deep learning to contribute to the diagnosis of AA has been investigated for a long time. In this study, CT images of patients who were pathologically confirmed to have AA were classified by taking into account the difficulties encountered by radiologists in diagnosis. Over 80% success was achieved using the deep learning technique in a patient group whose appendix diameter and peripheral edema, which are the most important clues for radiologists in diagnosis, were almost similar to a non-AA control group. In another group of patients with a significant diameter increase and significant peripheral edema in which radiologists did not have difficulties making a diagnosis, the diagnostic success rate with deep learning was almost 100%. This success rate can be further increased by increasing the number of patients. In addition, research can be conducted using deep learning techniques in cases where disassembly cannot be achieved due to superposition, a circumstance in which radiologists encounter difficulties in identifying the appendix.

## Figures and Tables

**Table 1:** Radiological, laboratory, and clinical findings.

		Group 1	Group 2	Control
Appendix double-wall thickness (mm)	IQR	0	4	1
	Mean (std)	7.8 (0.4)	12.1 (2.7)	6.6 (1.18)
	Min	6	6	4
	Max	9	20	10
WBC	IQR	5.1	5	2
	Mean (std)	5.6 (12.6)	14.5 (3.91)	5.7 (1.37)
	Min	3.9	1.2	4
	Max	22.8	25.1	9
CRP	IQR	51.7	89.8	1
	Mean (std)	37.4 (52.3)	63.5 (77)	3.5 (0.9)
	Min	3.1	6.3	1
	Max	255.3	409	5
Lower right quadrant pain		Yes	Yes	No
Age		14–65	13–68	13–67
Sex		Male: 34 Female: 32	Male: 103 Female: 97	Male: 145 Female: 121
Radiologist CT report		Normal (20) Definite AA (18) Suspicious (28)	Definite AA	Normal
Rebound		Yes	Yes	N/A
Defense		Yes	Yes	N/A
Peri-appendiceal edema		None/Minimal	Significant	N/A

WBC—white blood cells; CRP—C-reactive protein. N/A—Not available.

**Table 2:** Comparison of the proposed model and other models.

	Sensitivity (%)	Specificity (%)	Precision (%)	F1 Score
Mobile Net	0.914	0.583	0.871	0.891
Vgg16	0.923	0.554	0.887	0.904
DenseNet	0.917	0.496	0.855	0.884
Hybrid	0.941	0.572	0.894	0.916
Hybrid + EL (Proposed)	0.957	0.697	0.928	0.942

**Table 3:** Sensitivity of the proposed model in AA groups.

	AA (TP)	Non-AA (FN)	Sensitivity
Group 1	55	11	83.3%
Group 2	192	8	96%

AA—acute appendicitis; TP—true positive; FN—false negative.

## Data Availability

The data of the research are available from the authors.
